# *In vitro* comparison of three common essential oils mosquito repellents as inhibitors of the Ross River virus

**DOI:** 10.1371/journal.pone.0196757

**Published:** 2018-05-17

**Authors:** Miora Ralambondrainy, Essia Belarbi, Wildriss Viranaicken, Renata Baranauskienė, Petras Rimantas Venskutonis, Philippe Desprès, Pierre Roques, Chaker El Kalamouni, Jimmy Sélambarom

**Affiliations:** 1 Université de la Réunion, UM 134 Processus Infectieux en Milieu Insulaire Tropical (PIMIT), INSERM U1187, CNRS UMR9192, IRD UMR249, Plateforme Technologique CYROI, Sainte Clotilde, France; 2 Université Paris-Sud, INSERM U1184, CEA, Immunology of Viral Infections and Autoimmune Diseases, Institut de Biologie François Jacob, Fontenay-aux-Roses, France; 3 Kaunas University of Technology, Department of Food Science and Technology, Kaunas, Lithuania; University of California, San Francisco, UNITED STATES

## Abstract

**Background:**

The essential oils of *Cymbopogon citratus* (CC), *Pelargonium graveolens* (PG) and *Vetiveria zizanioides* (VZ) are commonly used topically to prevent mosquito bites and thus the risk of infection by their vectored pathogens such as arboviruses. However, since mosquito bites are not fully prevented, the effect of these products on the level of viral infection remains unknown.

**Objectives:**

To evaluate *in vitro* the essentials oils from Reunion Island against one archetypal arbovirus, the Ross River virus (RRV), and investigate the viral cycle step that was impaired by these oils.

**Methods:**

The essential oils were extracted by hydrodistillation and analyzed by a combination of GC-FID and GC×GC-TOF MS techniques. *In vitro* studies were performed on HEK293T cells to determine their cytotoxicity, their cytoprotective and virucidal capacities on RRV-T48 strain, and the level of their inhibitory effect on the viral replication and residual infectivity prior, during or following viral adsorption using the reporter virus RRV-*ren*Luc.

**Results:**

Each essential oil was characterized by an accurate quantification of their terpenoid content. PG yielded the least-toxic extract (CC_50_ > 1000 μg.mL^-1^). For the RRV-T48 strain, the monoterpene-rich CC and PG essential oils reduced the cytopathic effect but did not display virucidal activity. The time-of-addition assay using the gene reporter RRV-*ren*Luc showed that the CC and PG essential oils significantly reduced viral replication and infectivity when applied prior, during and early after viral adsorption. Overall, no significant effect was observed for the low monoterpene-containing VZ essential oil.

**Conclusion:**

The inhibitory profiles of the three essential oils suggest the high value of the monoterpene-rich essential oils from CC and PG against RRV infection. Combined with their repellent activity, the antiviral activity of the essential oils of CC and PG may provide a new option to control arboviral infection.

## Introduction

Management of arthropod-borne viruses (arboviruses) related to neglected tropical diseases has become a global health public concern [1]. The discovery of novel prophylactic or therapeutic treatments against arboviruses remain a continuous goal aimed to counter emerging virus or new viral strains [2]. Ross River virus (RRV) is a small enveloped positive-sense single-stranded RNA virus that belongs to the alphavirus genus, family *Togarividae* [3]. The RRV incubation period in humans was estimated to be from 7 to 9 days [4] and like the well-known Chikungunya virus that belongs to the same viral family, it typically causes fever, rash and polyarthralgia [5, 6]. RRV is endemic in Australia where it is the most common mosquito-borne pathogen with an average of 5000 cases annually [7, 8]. After the major outbreak in the Pacific area in 1979 and 1980, serological studies revealed the silent circulation of RRV in the Fiji islands [9] and more recently in French Polynesia [10, 11].

Two biological characteristics distinguish RRV from other alphaviruses: more than 40 species of mosquitoes can act as its vectors, thus, providing a large number of potential amplification cycles, and numerous warm blood host (mainly marsupials) support this virus’s replication [12]. This provides numerous opportunities for RRV to infect humans and initiate outbreak foci [13]. Indeed, during 2017, a large outbreak was observed in the South-West Region of Australia with more than 2 thousand cases reported in less than 2 months. Because the infection lead to very painful and debilitating joint, up to months after the initial onset, the disease has a direct impact on health services and calls for direct responses from the Australian authorities [14]. Consequently, RRV for which no efficient treatment is available, remains a major focus of basic research, and necessitates on-going surveys by the Australian health services [13, 15–18]. Mannose binding lectin (MBL) has been proposed as an efficient therapeutic target to alleviate RRV-induced arthritis but to date only pentosan sulfate, initially approved for the treatment of cystitis in U.S., is available [19, 20]. In an *in vitro* re-evaluation of 40 plants species used in Australian folk medicine, inhibition of RRV-induced cytopathic effect (25–50%) was observed with the ethanolic extract of *Myoporaceae* and *Pittosporaceae* species [21]. Essential oils are natural complex mixtures and their antiviral properties are due to complementary and overlapping mechanisms, as assumed for herpes simplex virus (HSV), influenza virus and yellow fever virus. To date, the anti-infective properties of essential oils, though of growing interest, have not been explored for RRV [22–24].

In arboviruses-related control measures, a number of essential oils are exploited as topical repellents to reduce the incidence of mosquito bites [25]. However, as yet these have not been investigated for antiviral activity at the site of infection, the skin, where they could be absorbed percutaneously. Such additional benefits of skin-applied essential oils may offer a great opportunity to control the early stages of infection, even when their repelling action fails. *Cymbopogon citratus*, *Vetiveria zizanioides* (family: Poaceae) and *Pelargonium graveolens* (family: Geraniaceae) are distributed worldwide and their essential oils (denoted hereafter as CC, VZ and PG, respectively), are readily available and have notable mosquito repellent properties [26]. The aim of the present study was to investigate the inhibitory effects *in vitro* of these three common essential oils at non-cytotoxic concentrations against RRV. We assessed their effects on both virus entry using the wild-type of RRV-T48 strain (RRV-T48) and viral replication using a recombinant RRV expressing *Renilla reniformis* luciferase (RRV-*ren*Luc).

## Materials and methods

### Plant material

Fresh leaves of *Cymbopogon citratus* (DC) Strapf and *Pelargonium graveolens* L’Hér were harvested in July 2014 and June 2015 in Reunion Island. Roots of *Vetiveria zizanioides* (L.) Nash were harvested in December 2015. All plant samples were kindly provided by the CAHEB (Coopérative Agricole des Huiles Essentielles de Bourbon), Le Tampon, Reunion Island.

### Essential oil isolation and analysis

Essential oils were extracted in triplicate from 2.5 kg of aerial part (PG and CC) or roots (ZV) by hydrodistillation during 3 h using a Clevenger-type apparatus. Essential oils were decanted from aqueous phase, dried over anhydrous sodium sulfate and filtered using Minisart filters (0.2 μm). Samples were then stored at 4°C in darkness. The chemical composition of the essential oils was quantified by gas chromatography-flame ionization detector (GC-FID) on *Clarus 500* gas chromatograph and identified by gas chromatography-time-of-flight-mass spectrometry on a GC×GC-TOF MS LECO Pegasus 4D system [27].

### Cell culture

Human embryonic kidney cell line HEK293T (ATCC) and the kidney epithelial cell line Vero (ATCC) were grown in Dulbecco’s modified Eagle’s medium (DMEM, Dutscher, Issy-les-Moulineaux, France) or modified Eagle’s medium (MEM, Dutscher) supplemented with 10% fetal bovine serum heat inactivated (FBS, Dutscher) and completed with 2 mmol.L^-1^ l-glutamine (Dutscher), 100 U.mL^-1^–0.1 mg.mL^-1^ penicillin-streptomycin (Dutscher), 1 mmol.L^-1^ sodium pyruvate (Dutscher) and 250 μg.mL^-1^ amphotericin (Dutscher). Cells were maintained in a humidified atmosphere of 5% CO_2_ at 37°C in Petri dishes.

### Cytotoxicity assay

Cell viability was measured using the tetrazolium salt MTT (3-(4,5-dimethylthiazol-2-yl)-2,5-diphenyltetrazolium bromide) assay [28]. Vero or HEK293T cells were seeded in 96-well plates (2×10^5^ cells/well) and allowed to adhere overnight at 37°C. Cells were then treated 21 h at 37°C with each essential oil within a wide range of concentrations (0.14–1130 μg.mL^-1^). The essential oils were solubilized in 0.4% DMSO (Sigma). Following the treatment, 20 μL of 5 mg.mL^-1^ MTT solution (Sigma, Saint-Quentin Fallavier France) were added on the 96-well plates and cells were stored at 37°C in the darkness. After 3 h of treatment, supernatant was removed and replaced by 100 μL of DMSO. Plates were then read at 570 nm on a microplate reader Tecan Sunrise™. The cytotoxic concentrations are defined as the concentration of the essential oil that causes death to 50% (CC_50_) or 10% (CC_10_) of viable cells with respect to controls without the essential oil (Table 1). Experiments were performed in hexaplicate from five independent experiments (see S1 Fig).

**Table 1:** Main characteristics of the essential oils.

Essential oil	Major components %	CC_50_ (μg.mL^-1)^^a^	CC_10_ (μg.mL^-1^)^b^
*Cymbopogon Citratus* (CC)	Geranial (45.11 ± 2.46%) Neral (32.16 ± 0.69%) Myrcene (7.85 ± 1.46%)	49.5 ± 20.5	17.6 ± 8.2
*Pelargonium Graveolens* (PG)	Citronellol (23.43± 0.14%) Geraniol (16.85 ± 0.05%) Citronellyl formate (12.29 ± 0.05%) Linalool (10.79 ± 0.05%) Isomenthone (7.06 ± 0.02%)	> 1000	533 ± 199
*Vetiveria Zizanoides* (VZ)	Khusimol (23.78 ± 0.13%) (*E*)-Isovalencenol (6.79 ± 0.12%) α-Vetivone (3.84 ± 0.04%)	169.9 ± 72.2	29.4 ± 13.5

^a^ Cytotoxic concentration CC_50_ that cause death to 50%

^b^ Cytotoxic concentration CC_10_ that causes death to 10%. Results are expressed as mean ± SD from triplicate determination.

### Virus strains

Ross River virus derived from an infectious clone of strain T48 (RRV-T48), GenBank GQ433359 was generously provided by Professor Richard J. Khun, Purdue University [29]. The recombinant RRV-T48 expressing *Renilla reniformis* luciferase, as an integral part of the non-structural polyprotein precursor, was obtained according to the procedure previously described for Chikungunya virus [30]. Briefly, the resulting plasmids pRRV-*ren*Luc was linearized and the corresponding RNA was transcribed *in vitro* using the kit mMESSAGE mMACHINEs Kit (Ambion) before transfection into Vero cells to provide live RRV-*ren*Luc viruses [31]. Supernatants were collected after 48 h (first passage) and used to infect Vero cells in order to grow final virus stocks for experiments (second passage).

### Plaque reduction assay

Virus titers were determined by plaque assays in Vero cells growing in 48-well plates as previously described [32]. Briefly, cells at confluence were incubated (0.1 mL/well) in triplicate with a serial of ten-fold dilutions of RRV-*ren*Luc containing samples for 2 h at 37°C. Then, 0.1 mL of 0.8% carboxymethylcellulose (CMC, Sigma) was added. After 48 h incubation at 37°C, CMC was removed and the monolayers were fixed with 3.7% v/v of paraformaldehyde (10 min at room temperature). Cells were then stained with 0.5% w/w crystal violet solution (10 min) and the virus titer in each well was estimated by counting the number of plaques observed. The results are expressed as plaque-formation unit per milliliter (PFU.mL^-1^).

### Cytoprotective effect of the essential oils against RRV-T48

The capacity of the tested essential oils on RRV-T48 inducing cytopathic effect was assessed on HEK293T cells using MTT assay as described above. HEK293T were seeded in 96-well plates (2×10^5^ cells/well) and allowed to adhere overnight at 37°C. Cells were infected by RRV-T48 alone at MOI 2 or by RRV-T48 at MOI 2 and addition of the essential oil at the highest non-toxic concentration (1×CC_10_) during 2 h at 37°C. After virus adsorption, supernatants were removed and cell culture medium or 1×CC_10_ of the essential oil was added again. Cell viability was measured at different time points from incubation at 37°C (6;12;18;24;30;36;42;48 or 24;32;48 h post-infection). Controls consist in RRV-infected-HEK293T cells untreated with the essential oils. Chloroquine at non-toxic concentration was used as positive control.

### Virucidal activity of the essential oils against RRV-T48 and entry assay

To determine the virucidal activity, each essential oil at concentrations 0.1×CC_10_ or 1×CC_10_ was mixed with the RRV-T48 strain (1×10^5^ PFU) during 1 h at 37°C. The residual infectivity was titrated by plaque assay on Vero cells with a serial of ten-fold dilutions. For the entry assay, Vero cells monolayers were pre-treated 3 h at 37°C with the essential oils. The residual infectivity of RRV-T48 strain was then titrated by plaque assay with a serial of ten-fold dilutions on the pre-treated Vero cells. All the experiments were performed in triplicate from five independent experiments and controls consist in RRV-T48-infected Vero cells without treatment with the essential oils.

### RRV-renLuc reporter assay

After infection with RRV-renLuc, the luciferase reporter is released in the cytoplasm during the polyprotein processing. HEK293T cells were seeded in 96well plates (2×10^5^ cells/well) and allowed to adhere overnight at 37°C. Cells were infected with RRV-*ren*Luc at MOI 2 for 48 h. At different time points (6;12;18;24;30;36;42;48 h post-infection), supernatants were removed and cells were lysed using 20 μL of lysis buffer (0.4% CHAPS, 10% glycerol, 1 mmol.L^-1^ EGTA, Tris-HCl, Sigma). 100 μL of the substrate coelenterazine (Euromedex, Souffelweyersheim, France) were then added and plates were immediately read by a luminescent plate-reader FLUOstar® Omega (BMG Labtech, Offenburg, Germany). Experiments were performed in hexaplicate from five independent experiments.

### Time-of-addition assay

To assess the effect of the essential oils on the replication of RRV, HEK293T cells were seeded in 96-well plates (2×10^5^ cells/well) and allowed to adhere overnight at 37°C. Cells were infected with RRV-*ren*Luc at MOI 2 in presence of the essential oil. The essential oils were added at the highest non-toxic concentration (1×CC_10_): (i) 3 h before virus incubation (pre-treatment) and after virus incubation; (ii) during virus incubation (co-treatment) and after virus incubation; (iii) 2 h, 4 h or 6 h after virus incubation (post-treatment). In all cases, the luciferase activity was measured in hexaplicate from five independent experiments by the RRV-*ren*Luc assay. Virus titers were determined from five independent experiments from harvested supernatants. The end point of these two analyses was 24 h post-infection. Controls consist in RRV-infected-HEK293T cells untreated with the essential oils. Chloroquine at non-toxic concentration was used as positive control.

### Statistical analysis

Where applicable, a one way analysis of variance (ANOVA) followed by Tukeys or Dunn’s post-test when relevant, using the GraphPad Prism software, version 7.01 (GraphPad Software Inc.). A *p* value lower than 0.05 was considered significant. Values are reported as the means standard errors (SEM) of n = 5 determinations unless otherwise stated. For the cytotoxicity assay, values were derived from dose-response curves and were calculated using GraphPad Prism.

## Results

### Chemical composition and cytotoxicity of essential oils

The combination of GC-FID and GC-TOF MS allowed identifying 37, 67 and 53 components of the essential oils from CC, PG and VZ, respectively, and their major components are listed in Table 1 (detailed chemical compositions are available in S1–S3 Tables). For the CC essential oil, the major components were the two isomeric monoterpene aldehydes geranial (45.11%) and neral (36.11%) and a high amount of the monoterpene hydrocarbon myrcene (7.85%) typical for essential oils of African origin [33]. The chemical composition of the PG oil was also found to be in accordance with the African type [34] with five major monoterpenes, including citronellol (23.43%), geraniol (16.85%) and linalool (10.79%) alcohols beside the citronellyl formate ester (12.29%) and isomenthone ketone (7.06%). The major components identified for the VZ extract include the sesquiterpenes khusimol (23.78%), (*E*)-isovalencenol (6.79%) and α-vetivone (3.84%), which are related to the Reunion chemotype [35]. The cytotoxic concentrations CC_50_ and CC_10_ of the three essential oils are reported in Table 1. The lowest CCs values were found for the CC essential oil followed by that of VZ, with the PG essential oil proving to be far less toxic (CC_50_> 1000 μg.mL^-1^; CC_10_ = 533 ± 199 μg.mL^-1^).

### Cytoprotective effect of the essential oils against RRV-T48

**Fig 1:**
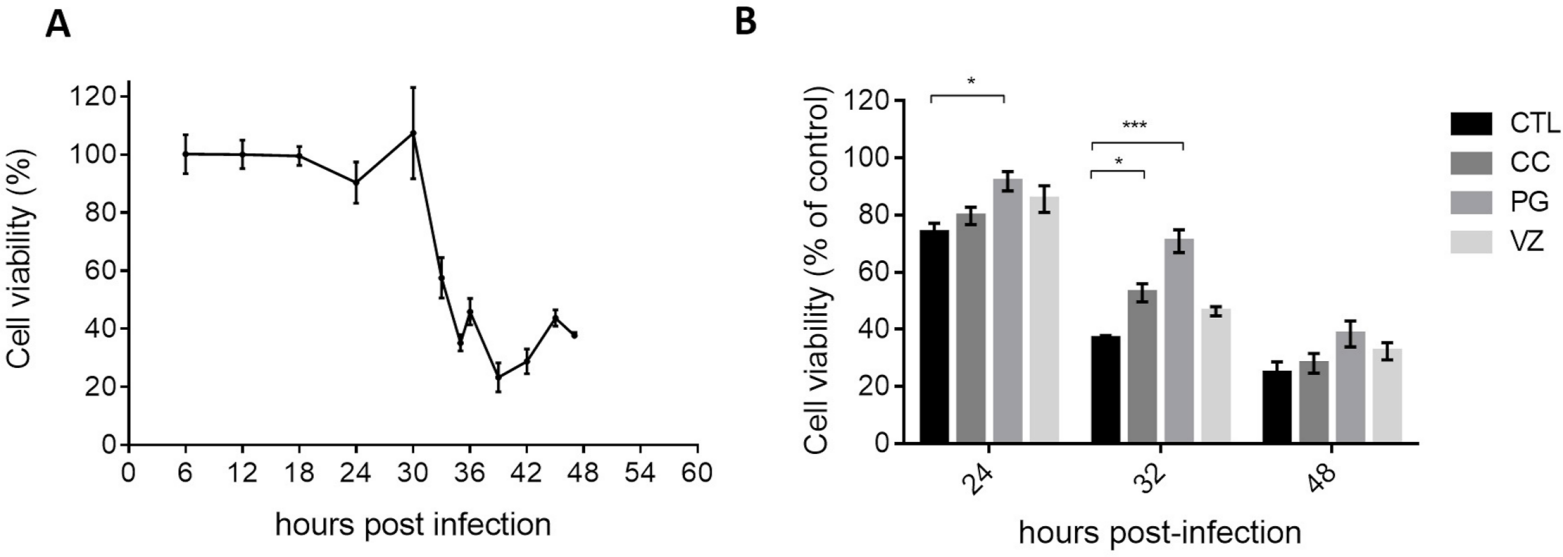
Viability of HEK293T cells infected with RRV-T48. **A:** without treatment by the essential oils; **B**: upon treatment with the essential oil at the concentration 1×CC_10_ at 24, 32 or 48 h post-infection. Controls (CTL) consist in untreated cells with the essential oils and results are expressed as mean ± SEM (n = 5). Statistical analysis was performed with Prism 7 (*p < 0.05, **p < 0.01, ***p < 0.005).

The antiviral activity of the essential oils was first screened by assessing the reduction of the viral cytopathic effect through determination of the viability of HEK293T cells by an MTT assay after infection by RRV-T48 at MOI 2. As shown in Fig 1, the viability of the infected cells without treatment was dramatically reduced beyond 30 h post-infection. Upon treatment by the essential oils at the non-toxic concentration 1×CC_10_, cell viability was determined at 24 h, 32 h or 48 h post-infection and compared with untreated cells. The results in Fig 1B showed that cell viability increases significantly at 32 h post-infection upon treatment with the essentials oils of CC (*p* < 0.01) or PG (*p* < 0.005). However, there was no more significant difference at 48 h post-infection.

### Virucidal activity of essential oils against RRV-T48 and entry assay

**Fig 2:**
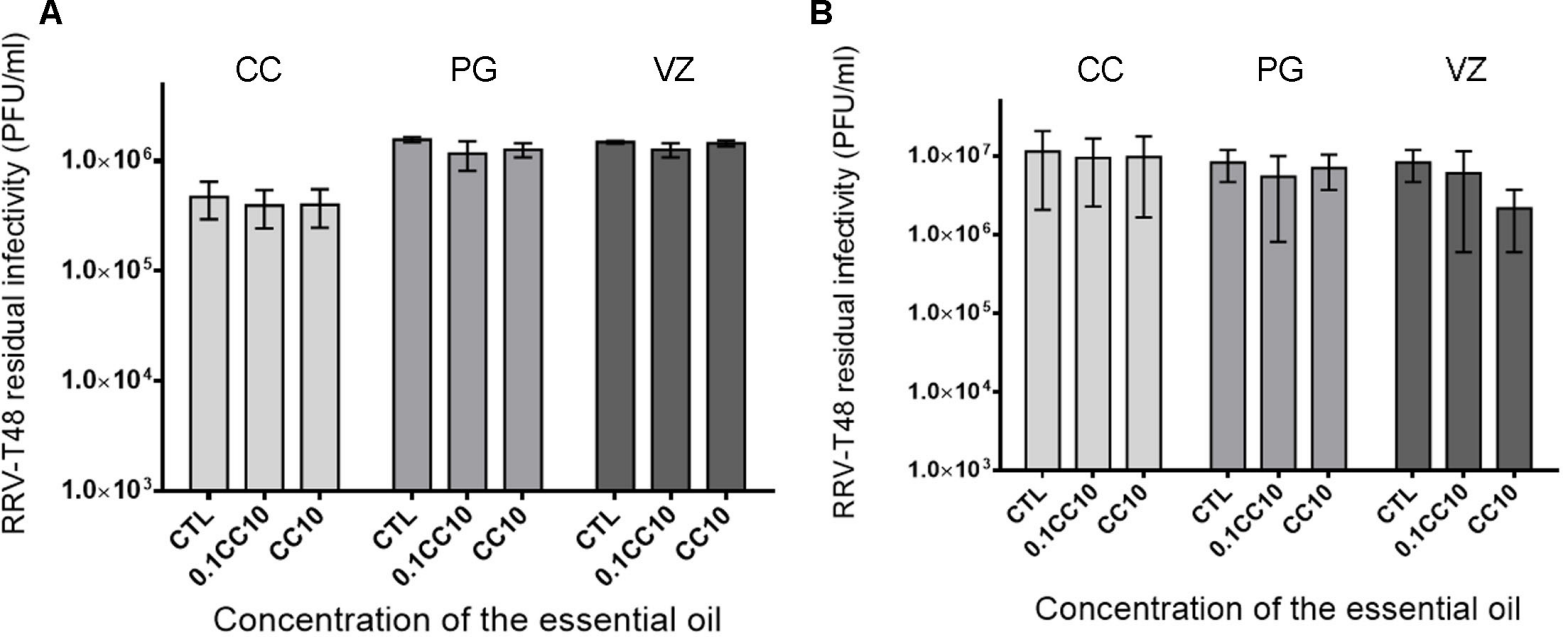
**Infectious capacity of RRV-T48** determined by plaque assay on infected Vero cells after pre-treatment of the viruses (1×10^5^ PFU) with the essential oils: **A:** residual infectivity; pre-treatment of the cells with the essential oils **B**: entry inhibition. Controls are infected cells and virus without treatment by the essential oils and values are expressed as mean ± SEM (n = 5).

In order to determine whether the essential oils interfere with virus entry, the residual infectivity was determined by plaque assay on Vero cells incubated with a mixture of virus (1×10^5^ PFU) and the essential oils (0.1×CC_10_ or 1×CC_10_). The infected cells without exposure to the essential oils were used as controls. As shown in Fig 2A, no significant effect was observed whichever essential oil was used. For the entry assay performed on cells pre-treated with the essential oils before infection, viral progeny production was not affected (Fig 2B).

### Inhibition of RRV-renLuc replication by the essential oils

**Fig 3:**
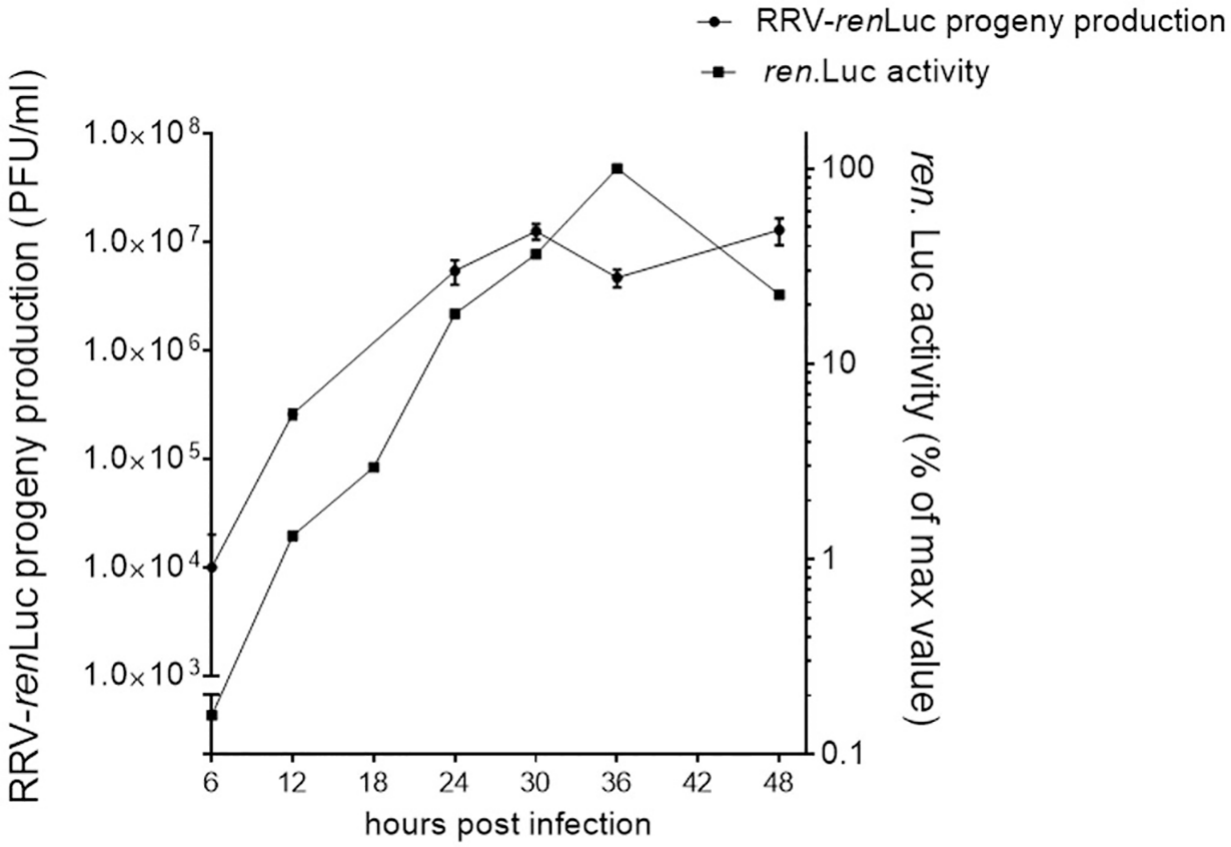
Time course of infection from RRV determined by plaque assay on infected Vero cells and luciferase activity on infected HEK293T cells. Results are expressed as a mean ± SEM (n = 5).

In order to determine the inhibitory effects of the essential oils at the early stage of the viral replication, we used a luciferase-based monitoring method using RRV-*ren*Luc and controlled the residual infectivity by plaque assay. The time course of infection of RRV-*ren*Luc (MOI 2) on HEK293T cells was first determined to guide subsequent experiments. We observed that the luciferase activity increases until 36 h post-infection (Fig 3) while the viral progeny production reaches a maximum at 30 h post-infection (Fig 3). Thus, the effect of essential oils on RRV-*ren*Luc replication levels was determined 24 h after infection.

**Fig 4:**
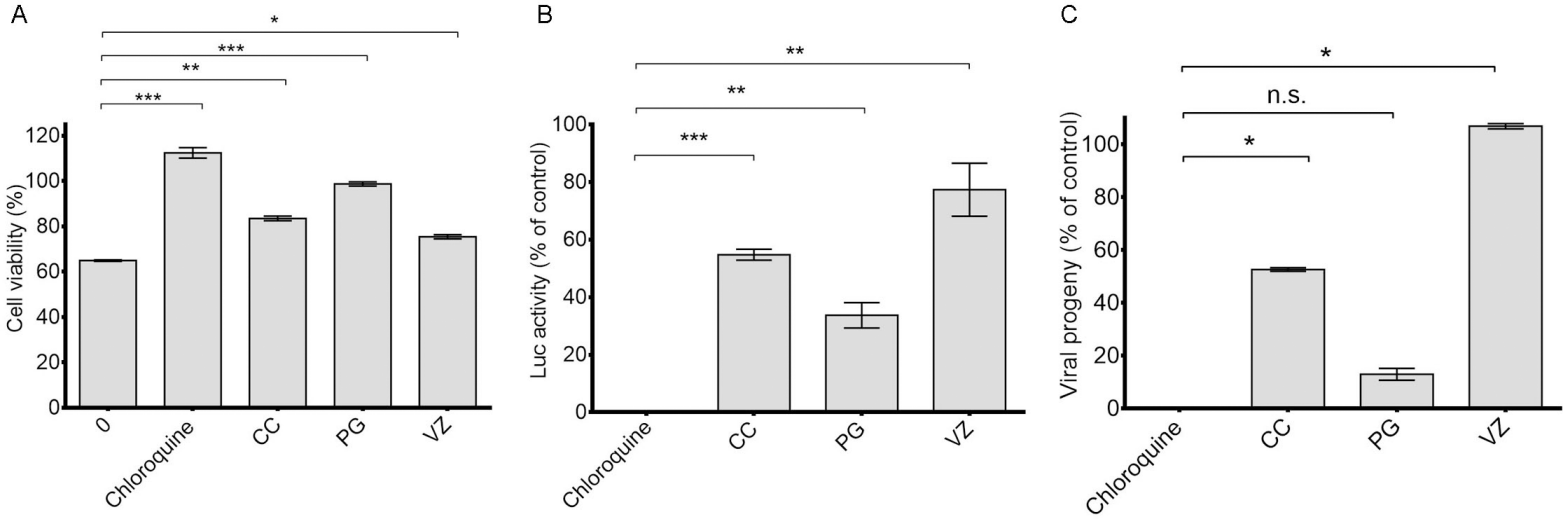
Comparative effect of the essential oils on HEK293T against RRV. Viability of HEK293T cells infected with RRV-T48 (MOI 2) upon treatment with chloroquine at the concentration 20 μg.mL^-1^and essential oil at the concentration 1× CC_10_ at 24 h post-infection (**A**); Inhibition of RRV-*ren*Luc replication at MOI 2 using co-treatment of chloroquine at the concentration 20 μg.mL^-1^and essential oil at the concentration 1× CC_10_ (**B**); Viral growth by plaque assay on Vero cells (**C**). Controls are RRV-infected cells without treatment by chloroquine and the essential oils. Values are expressed as mean ± SEM (n = 5). (*p < 0.05, **p < 0.01, ***p < 0.005).

In a preliminary assessment, we compared the inhibitory capacity of the essential oils with that of the broad-range viral replication inhibitor chloroquine [36]. As shown in Fig 4A, at the non-toxic concentration 1×CC_10_ for the essential oils and for chloroquine, cell viability was not affected. Maximal reduction of luciferase activity and residual infectivity was observed for the PG essential oil (Fig 4B and 4C).

**Fig 5:**
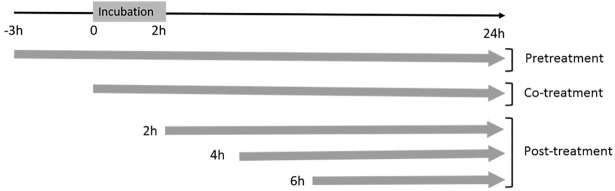
Time points of essentials oils addition and end point of analysis using RRV-*ren*Luc infection at MOI 2 on HEK293T cells.

To assess if the inhibitory effects of the essential oils is related to the viral absorption or entry we altered treatment timing (pre-treatment, co-treatment or post-treatment, Fig 5).

**Fig 6:**
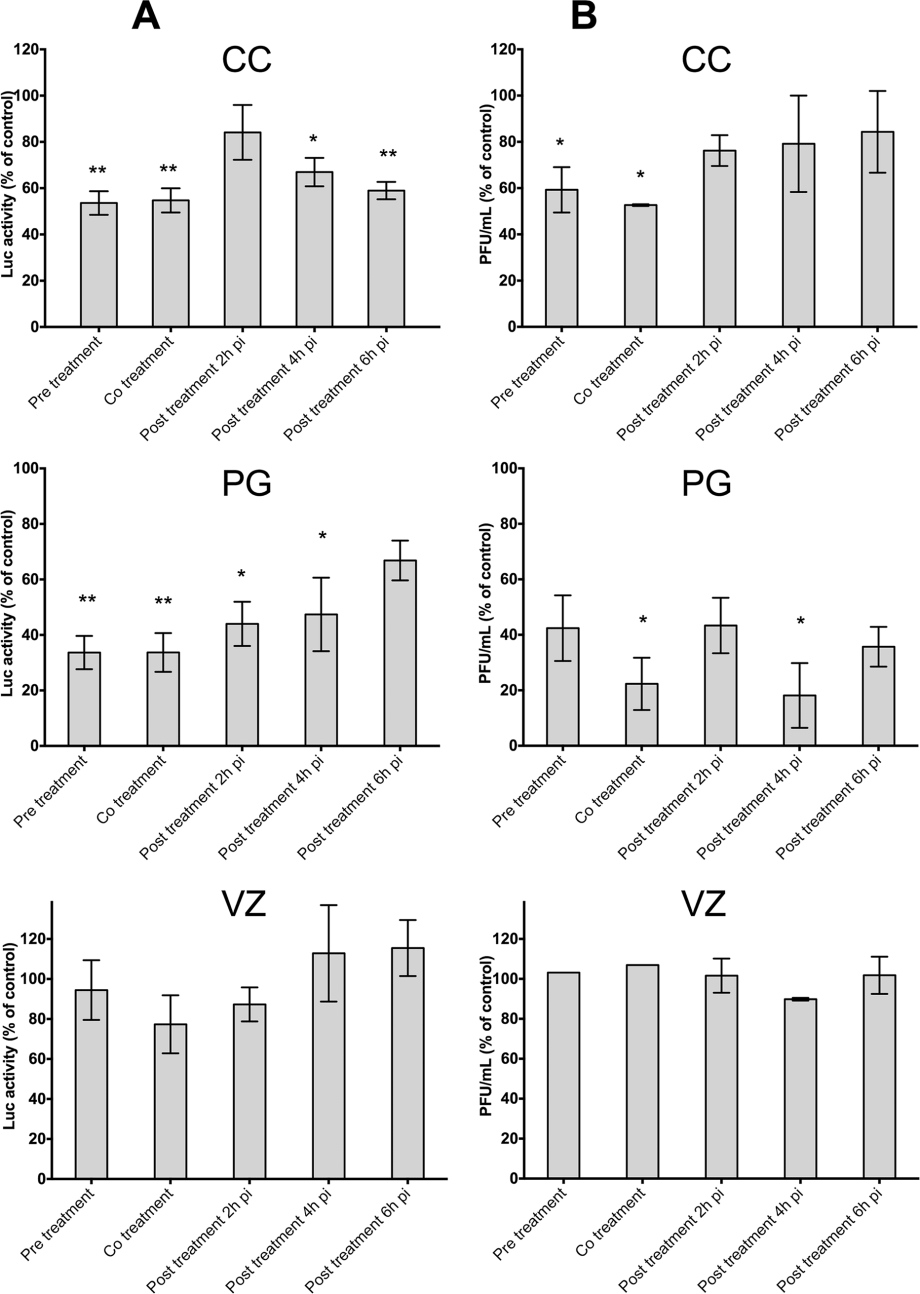
Inhibition of RRV-*ren*Luc replication by the 3 essential oils under the experimental conditions shown in Fig 5. HEK293T was exposed to RRV-*ren*Luc at MOI 2 and treated as shown in Fig 5 with essential oils (**A**) luciferase activity on HEK293T cells (**B**) Viral growth by plaque assay on Vero cells. Controls are RRV-*ren*Luc-infected cells infected without treatment by the essential oils. Values are expressed as relative percentage ± SEM (n = 5). (*p < 0.05, **p < 0.01, ***p < 0.005).

The overall results are presented in Fig 6. Using the CC essential oil, only pretreatment and co-treatment significantly decrease both luciferase activity and residual infectivity titer. The maximum inhibitory effect was observed for co-treatment, with both a luciferase activity level and a virus residual infectivity titer down close to 50% of the non-treated (p ≤ 0.01 and p≤ 0.05 respectively). For the PG essential oil, all treatments induced a significant decrease of the virus activity with the lowest level of luciferase activity observed for pre-treatment (34%) but later was the treatment, more increased the luciferase activity to reach a high level (67%) for post-treatment at 6 h post-infection (ANOVA one-way test for trend slope = 13.2, p<0.001). However, the reduction in virus residual infectivity titer is always significant (p<0.05 to 0.01) compared to non-treated control, it varied in a non-regular manner not significantly different whatever was the timing of treatment (p>0.058, Kruskal-Wallis test) even if maximum reduction was observed by co-treatment and post-treatment at 4 h post-infection (< 20%). In contrast, the VZ essential oil exhibited no significant effect either on luciferase activity or on viral progeny production whatever was the treatment timing. Thus, the inhibitory profiles were found to be significantly different for the three tested essential oils.

## Discussion

Essential oils have demonstrated a wide range of biological activities (e.g. antibacterial, antifungal, antioxidants, etc…) and remain promising sources of new therapeutics [37, 38]. A growing attention has been given to the antiviral capacity of essential oils against arboviruses as illustrated for dengue serotype 2 (DEN-2) virus [39], Yellow Fever virus (YFV) [40], and Japanese Encephalitis virus (JEV) [41]. To the best of our knowledge, none of the available essential oils used as topical mosquito repellents have not yet been investigated for their antiviral capacity against mosquito-borne viruses. In here, we investigated such inhibitory effects of three common essential oils of CC, PG and VZ at non-toxic concentrations against RRV infection.

For this study, HEK293T cells were selected for their high sensitivity to the viral cytopathic effect, in contrast to the resistant skin cell lines [42]. In addition, we used a representative RRV strain to provide a fast and representative evaluation of essential oils against the alphaviruses family.

The cytopathic effect of RRV-T48 on HEK293T cells observed after 30 h post-infection (Fig 1A) and the time-limited cytoprotective effect of the tested essential oils at 32 h post-infection clearly indicated the best opportunity to control RRV infection at the early stages. Specific assays (Fig 2) showed that the essential oils do not have virucidal activity and could not interfere with virus entry at the two non-toxic concentrations tested (0.1×CC_10_ and 1×CC_10_). Thus, we carried out further investigations to determine the inhibitory effect of the essential oil on viral replication. This was supported by the time course of infection of RRV-*ren*Luc at MOI 2 that reached a maximum at 36 hours (Fig 3A), leading us to select the endpoint of monitoring at 24 h post-infection for both the luciferase activity and the residual infectivity upon different treatments by the essential oils (Fig 5).

Interestingly, the time-of-addition assay showed that the additional supply of the essential oils of CC and PG prior or during or the viral adsorption (pre-treatment or co-treatment) provides the most significant inhibition of the viral replication (Fig 6A). The viral replication was also reduced in post-treatment (Fig 6A), but only the PG essential oil exhibited a marked effect on the residual infectivity (Fig 6B). This latter result suggests that the PG essential oil may also interfere with the post-transcriptional stage of the virus life cycle.

Thus, the results showed the high potential of the PG essential oil against RRV, as it has low cytotoxicity (CC_50_ > 1000 μg.mL^-1^) and displays noteworthy inhibitory effects when present prior, during or after infection. The CC essential oil, which presented the highest cytotoxicity (CC_50_ = 49.5 ± 20.5 μg.mL^-1^), exhibited a moderate antiviral activity when introduced prior or during the viral absorption. In contrast, there was no evidence for an inhibitory effect from the VZ essential oil. The contribution of the major components of essential oils to their antiviral properties has been claimed in the case of HSV-1 [43–45]. PG and CC essential oils are monoterpene-rich essential oils in contrast to the non-active VZ essential oil that is mainly constituted by sesquiterpenes. Thus, we propose that antiviral capacity is related to the monoterpene composition, and the actual molecular components deserve to be characterized and explored further.

## Conclusion

The repellent activity of the three essential oils from CC, PG and VZ was known from folk medicine. The present study provides the first investigation of their antiviral activity against RRV infection. The different inhibitory profiles of these three essential oils suggest a relationship with their chemical compositions. Exposure to these oils prior or at the same time as viral infection resulted in the highest inhibitory effects and suggest that the primary application of these essential oils as repellents may further provide an additional valuable preventive effect against the viral infection. Our findings demonstrate the value of re-evaluating essential oils mosquito repellents for their antiviral capacity, as this might provide a novel, eco-friendly and cost-effective strategy in the prevention of arboviruses infection.

## Supporting information

S1 Fig Determination of essential oils cytotoxicity on HEK293T. Viability of HEK293T cells was determined by MTT assay upon treatment by the essential oils of CC (A); PG (B); VZ (C). Values are expressed as mean ± SEM (n = 3). Dashed line indicated the CC_10_.
(DOCX)

S1 Table Chemical composition of the leaf *Cymbopogon citratus* (CC) essential oil from Reunion Island, area percentage mean ± standard deviation (n = 9). (DOCX)

S2 Table Chemical composition of the leaf *Pelargonium graveolens* (PG) essential oil from Reunion Island, area percentage mean ± standard deviation (n = 9). (DOCX)

S3 Table Chemical composition of the roots *Vetiveria zizanioides* (ZV) essential oil from Reunion Island, area percentage mean ± standard deviation (n = 6). (DOCX)
