# Supplementary material for: In vitro comparison of three common essential oils mosquito repellents as inhibitors of the Ross River virus
Source: PLoS One. 2018 May 17;13(5):e0196757. doi: 10.1371/journal.pone.0196757 (PMC5957362; doi:10.1371/journal.pone.0196757)
Supplement: S1 Fig — Viability of HEK293T cells was determined by MTT assay upon treatment by the essential oils of CC (A); PG (B); VZ (C). Values are expressed as mean ± SEM (n = 3). Dashed line indicated the CC10. (DOCX) [file pone.0196757.s001.docx]

Supplementary Figure 1


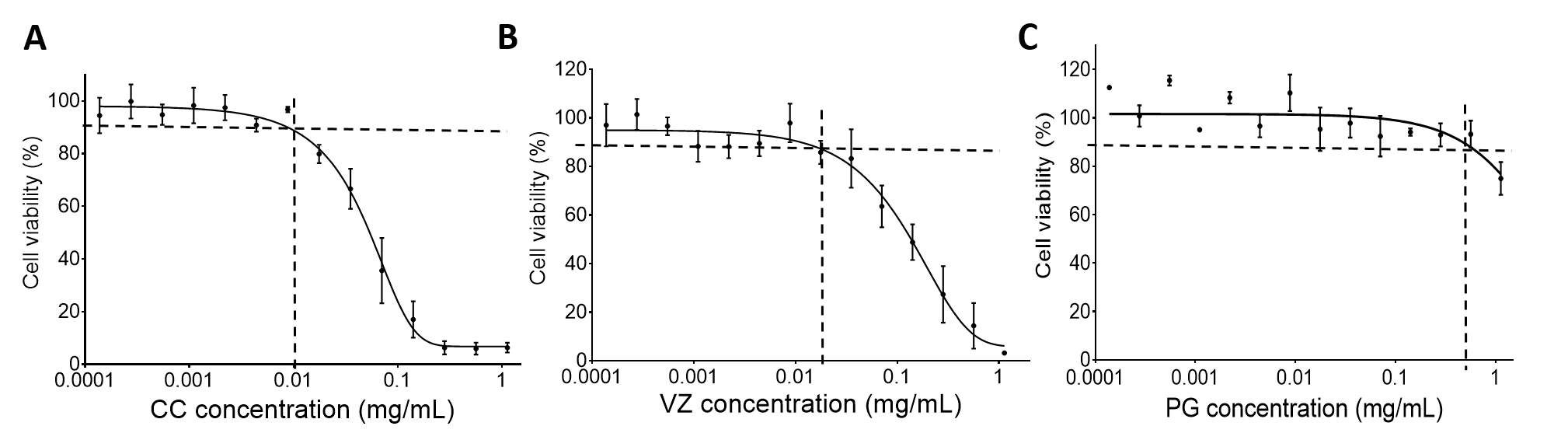


**Fig. 1. Determination of essential oils cytotoxicity on HEK293T.** Viability of HEK293T cells was determined by MTT assay upon treatment by the essential oils of CC (A); PG (B); VZ (C). Values are expressed as mean ± SEM (n = 3). Dashed line indicated the CC_10_.
