# Supplementary material for: In vitro comparison of three common essential oils mosquito repellents as inhibitors of the Ross River virus
Source: PLoS One. 2018 May 17;13(5):e0196757. doi: 10.1371/journal.pone.0196757 (PMC5957362; doi:10.1371/journal.pone.0196757)
Supplement: S1 Table — (DOCX) [file pone.0196757.s002.docx]

Supplementary Table 1

S1 Table. Chemical composition of the leaf *Cymbopogon citratus* (CC) essential oil from Reunion Island, area percentage mean ± standard deviation (n=9).

| No | Name | KI^a^ | KI^b^ | Identification | % |
| --- | --- | --- | --- | --- | --- |
| 1 | 6-Methyl-5-hepten-2-one | 988 | 985 | KI, MS | 0.76 ± 0.25 |
| **2** | **Myrcene** | **991** | **990** | KI, MS | **7.85 ± 1.46** |
| 3 | 1,8-Cineole | 1031 | 1031 | KI, MS | 0.04 ± 0.00 |
| 4 | (Z)-β-Ocimene | 1039 | 1037 | KI, MS | 0.33 ± 0.06 |
| 5 | (E)-β-Ocimene | 1050 | 1050 | KI, MS | 0.22 ± 0.04 |
| 6 | γ-Terpinene | 1062 | 1059 | KI, MS | 0.05 ± 0.00 |
| 7 | Terpinolene | 1094 | 1088 | KI, MS | 0.04 ± 0.00 |
| 8 | 6,7-Epoxymyrcene | 1098 | 1092 | KI, MS | 0.14 ± 0.04 |
| 9 | Linalool | 1100 | 1096 | KI, MS | 0.70 ± 0.10 |
| 10 | (Z)-Limonene oxide | 1143 | 1136 | KI, MS | 0.08 ± 0.03 |
| 11 | (Z)-Verbenol | 1148 | 1141 | KI, MS | 1.34 ± 0.17 |
| 12 | Citronellal | 1155 | 1153 | KI, MS | 0.60 ± 0.14 |
| 13 | β-Pinene oxide | 1161 | 1159 | KI, MS | 0.05 ± 0.01 |
| 14 | (Z)-Isocitral | 1167 | 164 | KI, MS | 1.61 ± 0.28 |
| **15** | **(E)-Isocitral** | **1184** | **1180** | KI, MS | **2.68 ± 0.47** |
| 16 | α-Terpineol | 1197 | 1188 | KI, MS | 0.08 ± 0.00 |
| 17 | Citronellol | 1233 | 1225 | KI, MS | 0.54 ± 0.11 |
| 18 | (Z)-Carveol | 1237 | 1229 | KI, MS | 0.07 ± 0.01 |
| **19** | **Neral** | **1249** | **1238** | KI, MS | **32.16 ± 0.69** |
| **20** | **Geraniol** | **1260** | **1252** | KI, MS | **3.29 ± 0.31** |
| **21** | **Geranial** | **1279** | **1267** | KI, MS | **45.11 ± 2.46** |
| 22 | Neryl formate | 1293 | 1282 | KI, MS | 0.03 ± 0.00 |
| 23 | 2-Undecanone | 1343 | 1368^c^ | KI, MS | 0.24 ± 0.15 |
| 24 | Neryl acetate | 1379 | 1361 | KI, MS | 0.34 ± 0.22 |
| 25 | Geranyl acetate | 1383 | 1381 | KI, MS | 0.49 ± 0.06 |
| 26 | β-Elemene | 1414 | 1390 | KI, MS | 0.08 ± 0.00 |
| 27 | Neric acid | 1424 | 1423^c^ | KI, MS | 0.05 ± 0.02 |
| 28 | (E)-Caryophyllene | 1427 | 1419 | KI, MS | 0.08 ± 0.01 |
| 29 | α-(E)-Bergamotene | 1438 | 1434 | KI, MS | 0.05 ± 0.01 |
| 30 | α-Humulene | 1458 | 1454 | KI, MS | 0.06 ± 0.00 |
| 31 | γ-Muurolene | 1493 | 1479 | KI | 0.04 ± 0.00 |
| 32 | Elemol | 1557 | 1549 | KI, MS | 0.26 ± 0.05 |
| 33 | Caryophyllene oxide | 1594 | 1583 | KI, MS | 0.08 ± 0.02 |
| 34 | τ-Muurolol | 1652 | 1642 | KI | 0.04 ± 0.00 |
| 35 | α-Cadinol | 1665 | 1654 | KI, MS | 0.09 ± 0.02 |
| 36 | Intermedeol | 1673 | 1666 | KI, MS | 0.12 ± 0.00 |
| 37 | Eudesm-7(11)-en-4-ol | 1709 | 1700 | KI, MS | 0.04 ± 0.00 |

^a^ Kováts retention indices calculated against C_7_–C_30_ *n*-alkanes on nonpolar Elite–5 column.

^b^ Kováts retention indices on nonpolar DB–5 column reported in literature (Adams, 2009).

^c^ Kpoviessi S., et al. (2014). Chemical composition, cytotoxicity and *in vitro* antitrypanosomal and antiplasmodial activity of the essential oils of four *Cymbopogon* species from Benin. Journal of Ethnopharmacology 151, 652-659.
