# Supplementary material for: In vitro comparison of three common essential oils mosquito repellents as inhibitors of the Ross River virus
Source: PLoS One. 2018 May 17;13(5):e0196757. doi: 10.1371/journal.pone.0196757 (PMC5957362; doi:10.1371/journal.pone.0196757)
Supplement: S2 Table — (DOCX) [file pone.0196757.s003.docx]

Supplementary Table 2

S2 Table. Chemical composition of the leaf *Pelargonium graveolens* (PG) essential oil from Reunion Island, area percentage mean ± standard deviation (n=9).

| No | Name | KI^a^ | KI^b^ | Identification | % |
| --- | --- | --- | --- | --- | --- |
| 1 | α-Pinene | 939 | 939 | KI, MS | 0.52 ± 0.01 |
| 2 | β-Pinene | 978 | 979 | KI, MS | 0.08 ± 0.00 |
| 3 | Myrcene | 994 | 990 | KI, MS | 0.71 ± 0.05 |
| 4 | dehydro-(E)-Linaloloxide | 997 | 993 | KI, MS | 0.10 ± 0.00 |
| 5 | α-Phellandrene | 1008 | 1002 | KI, MS | 0.09 ± 0.01 |
| 6 | *p*-Cymene | 1030 | 1024 | KI, MS | 0.36 ± 0.02 |
| 7 | Limonene | 1034 | 1029 | KI, MS | 0.14 ± 0.03 |
| 8 | β-Phellandrene | 1040 | 1029 | KI, MS | 0.08 ± 0.01 |
| 9 | (Z)-β-Ocimene | 1044 | 1037 | KI, MS | 0.23 ± 0.01 |
| 10 | (E)-β-Ocimene | 1054 | 1050 | KI, MS | 0.35 ± 0.02 |
| 11 | (Z)-Linaloloxide | 1080 | 1072 | KI, MS | 0.35 ± 0.00 |
| 12 | (E)-Linaloloxide | 1095 | 1086 | KI, MS | 0.19 ± 0.01 |
| **13** | **Linalool** | **1107** | **1096** | **KI, MS** | **10.79 ± 0.05** |
| 14 | (E)-Sabinene hydrate | 1111 | 1098 | KI, MS | 0.08 ± 0.01 |
| 15 | 6-Methyl-3,5-heptadiene-2-one | 1114 | 1105 | KI, MS | 0.07 ± 0.01 |
| 16 | (Z)-Rose oxide | 1117 | 1108 | KI, MS | 0.54 ± 0.01 |
| 17 | (E)-Rose oxide | 1136 | 1125 | KI, MS | 0.22 ± 0.01 |
| 18 | (Z)-3-Hexenyl isobutanoate | 1152 | 1146 | KI, MS | 0.07 ± 0.00 |
| 19 | Menthone | 1162 | 1152 | KI, MS | 0.46 ± 0.03 |
| **20** | **Isomenthone** | **1174** | **1162** | **KI, MS** | **7.06 ± 0.02** |
| 21 | Terpinen-4-ol | 1186 | 1177 | KI, MS | 0.07 ± 0.01 |
| 22 | *p*-Cymen-8-ol | 1189 | 1182 | KI, MS | 0.16 ± 0.01 |
| 23 | Isomenthol | 1191 | 1182 | KI, MS | 0.12 ± 0.00 |
| 24 | α-Terpineol | 1198 | 1188 | KI, MS | 0.83 ± 0.05 |
| 25 | Myrtenol | 1205 | 1195 | KI, MS | 0.07 |
| **26** | **Citronellol** | **1241** | **1225** | **KI, MS** | **23.43 ± 0.14** |
| 27 | Neral | 1251 | 1238 | KI, MS | 0.60 ± 0.00 |
| **28** | **Geraniol** | **1268** | **1252** | **KI, MS** | **16.85 ± 0.05** |
| 29 | (E)-Myrtanol | 1273 | 1261 | KI, MS | 0.30 ± 0.05 |
| 30 | Geranial | 1280 | 1267 | KI, MS | 0.86 ± 0.06 |
| **31** | **Citronellyl formate** | **1283** | **1273** | **KI, MS** | **12.29 ± 0.05** |
| 32 | Neryl formate | 1288 | 1282 | KI, MS | 0.16 ± 0.00 |
| **33** | **Geranyl formate** | **1309** | **1298** | **KI, MS** | **4.72 ± 0.04** |
| 34 | α-Cubebene | 1316 | 1351 | KI, MS | 0.21 ± 0.00 |
| 35 | Citronellyl acetate | 1359 | 1352 | KI, MS | 0.28 ± 0.01 |
| 36 | 2-Phenyl ethyl propanoate | 1363 | 1354 | KI, MS | 0.08 ± 0.01 |
| 37 | α-Ylangene | 1384 | 1375 | KI, MS | 0.14 ± 0.01 |
| 38 | α-Copaene | 1390 | 1376 | KI, MS | 0.40 ± 0.00 |
| 39 | β-Bourbonene | 1394 | 1388 | KI, MS | 0.46 ± 0.01 |
| 40 | Phenyl ethyl isobutanoate | 1399 | 1393 | KI, MS | 0.20 ± 0.00 |
| 41 | **(E)-Caryophyllene** | 1431 | 1419 | KI, MS | 0.84 ± 0.01 |
|  |  |  |  |  |  |
| **Table 2.** *(Continued)* | | | | | |
| No | Name | KI^a^ | KI^b^ | Identification | % |
| 42 | α-Guaiene | 1449 | 1439 | KI, MS | 0.58 ± 0.01 |
| **43** | **Guaia-6,9-diene** | **1455** | **1444** | **KI, MS** | **3.54 ± 0.05** |
| 44 | Citronellyl propanoate | 1460 | 1446 | KI, MS | 0.30 ± 0.01 |
| 45 | α-Humulene | 1466 | 1454 | KI, MS | 0.21 ±0.01 |
| 46 | allo-Aromadendrene | 1474 | 1460 | KI, MS | 0.11 ± 0.01 |
| **47** | **Geranyl propanoate** | **1481** | **1477** | **KI, MS** | **1.16 ± 0.02** |
| 48 | γ-Muurolene | 1487 | 1479 | KI, MS | 0.08 ± 0.01 |
| 49 | Germacrene | 1492 | 1481 | KI, MS | 0.60 ± 0.01 |
| 50 | Citronellol isobutanoate | 1498 | 1483 | KI, MS | 0.11 ± 0.00 |
| 51 | α-Selinene | 1511 | 1498 | KI, MS | 0.09 ± 0.01 |
| 52 | α-Muurolene | 1519 | 1500 | KI, MS | 0.24 ± 0.00 |
| 53 | δ-Amorphene | 1526 | 1512 | KI, MS | 0.09 ± 0.00 |
| 54 | (E)-Calamenene | 1534 | 1522 | KI, MS | 0.51 ± 0.00 |
| 55 | Citronellyl butanoate | 1535 | 1531 | KI, MS | 0.29 ± 0.00 |
| 56 | Furopelargone A | 1554 | 1540 | KI, MS | 0.28 ± 0.01 |
| **57** | **Geranyl butanoate** | **1567** | **1564** | **KI, MS** | **1.18 ± 0.01** |
| **58** | **2-Phenyl ethyl tiglate** | **1598** | **1585** | **KI, MS** | **1.03 ± 0.01** |
| 59 | Geranyl isovalerate | 1614 | 1607 | KI, MS | 0.09 ± 0.00 |
| 60 | 1,10-di-epi- Cubenol | 1629 | 1619 | KI, MS | 0.09 ± 0.01 |
| 61 | 10-epi-γ-Eudesmol | 1632 | 1623 | KI, MS | 0.20 ± 0.01 |
| 62 | Citronellyl valerate | 1637 | 1625 | KI, MS | 0.10 ± 0.00 |
| 63 | α-Muurolol | 1659 | 1646 | KI, MS | 0.27 ± 0.01 |
| 64 | β-Eudesmol | 1663 | 1650 | KI, MS | 0.15 ± 0.03 |
| 65 | Geranyl valerate | 1673 | 1656 | KI, MS | 0.50 ± 0.06 |
| 66 | Citronellyl tiglate | 1689 | 1668 | KI, MS | 0.12 ± 0.03 |
| **67** | **Geranyl tiglate** | **1709** | **1696** | **KI, MS** | **1.30 ± 0.02** |

^a^ Kováts retention indices calculated against C_7_–C_30_ *n*-alkanes on nonpolar Elite–5 column.

^b^ Kováts retention indices on nonpolar DB–5 column reported in literature (Adams, 2009).
