# Supplementary material for: In vitro comparison of three common essential oils mosquito repellents as inhibitors of the Ross River virus
Source: PLoS One. 2018 May 17;13(5):e0196757. doi: 10.1371/journal.pone.0196757 (PMC5957362; doi:10.1371/journal.pone.0196757)
Supplement: S3 Table — (DOCX) [file pone.0196757.s004.docx]

Supplementary Table 3

**S3 Table.** Chemical composition of the roots *Vetiveria zizanioides* (ZV) essential oil from Reunion Island, area percentage mean ± standard deviation (n=6).

| No |  | Name | KI^a^ | KI^b^ | Identification | % |
| --- | --- | --- | --- | --- | --- | --- |
| 1 |  | 1,8-Cineole | 1039 | 1031 | KI, MS | 0.08 ± 0.00 |
| 2 |  | Linalool | 1105 | 1096 | KI, MS | 0.84 ± 0.24 |
| 3 |  | Menthone | 1162 | 1152 | KI, MS | 0.10 ± 0.00 |
| 4 |  | Isomenthone | 1173 | 1162 | KI, MS | 0.07 ± 0.02 |
| 5 |  | Borneol | 1176 | 1169 | KI, MS | 0.06 |
| 6 |  | Terpinen-4-ol | 1185 | 1177 | KI, MS | 0.34 ± 0.08 |
| 7 |  | α-Terpineol | 1198 | 1188 | KI, MS | 0.42 ± 0.15 |
| **8** |  | **Citronellol** | **1237** | **1225** | **KI, MS** | **3.70 ± 1.42** |
| 9 |  | Geraniol | 1263 | 1252 | KI, MS | 0.52 ± 0.13 |
| 10 |  | α-Ylangene | 1375 | 1375 | KI, MS | 0.09 ± 0.02 |
| 11 |  | α-Copaene | 1378 | 1376 | KI, MS | 0.69 ± 0.03 |
| 12 |  | Isoledene | 1390 | 1376 | KI, MS | 0.07 ± 0.01 |
| 13 |  | β-Cubebene | 1392 | 1388 | KI, MS | 0.09 ± 0.01 |
| 14 |  | 7-epi-sesquithujene | 1401 | 1391 | KI, MS | 0.16 ± 0.11 |
| 15 |  | Acora-3,7(14)-diene | 1420 | 1408 | KI, MS | 0.07 ± 0.01 |
| 16 |  | β-Funebrene | 1424 | 1414 | KI, MS | 0.14 ± 0.04 |
| **17** |  | **2,5-dimethoxy*-p*-cymene** | **1433** | **1426** | **KI, MS** | **1.27 ± 0.28** |
| 18 |  | Prezizaene | 1460 | 1446 | KI, MS | 0.69 ± 0.12 |
| 19 |  | Khusimene | 1465 | 1455 | KI, MS | 0.89 ± 0.15 |
| 20 |  | α-Amorphene | 1493 | 1483 | KI, MS | 0.40 ± 0.05 |
| 21 |  | α-Vetispirene | 1496 | 1490 | KI, MS | 0.73 ± 0.13 |
| 22 |  | β-Vetispirene | 1502 | 1493 | KI, MS | 0.45 ± 0.24 |
| 23 |  | δ-Selinene | 1504 | 1492 | KI, MS | 0.12 ± 0.08 |
| 24 |  | δ-Amorphene | 1519 | 1512 | KI, MS | 0.10 ± 0.02 |
| 25 |  | Nootkatene | 1525 | 1518 | KI, MS | 0.13 ± 0.07 |
| 26 |  | δ-Cadinene | 1535 | 1523 | KI, MS | 0.28 ± 0.04 |
| 27 |  | γ-Vetivenene | 1542 | 1533 | KI, MS | 0.13 ± 0.04 |
| 28 |  | α-Calacorene | 1558 | 1545 | KI, MS | 0.69 ± 0.03 |
| 29 |  | Elemol | 1563 | 1549 | KI, MS | 0.54 ± 0.08 |
| 30 |  | β-Vetivenene | 1568 | 1555 | KI, MS | 0.28 ± 0.02 |
| **31** |  | **epi-Longipinanol** | **1577** | **1563** | **KI, MS** | **1.41 ± 0.02** |
| 32 |  | β-Calacorene | 1581 | 1565 | KI, MS | 0.15 ± 0.01 |
| 33 |  | Caryophyllene oxide | 1589 | 1583 | KI, MS | 0.13 ± 0.06 |
| 34 |  | 2-Phenyl ethyl tiglate | 1599 | 1585 | KI, MS | 0.63 ± 0.02 |
| 35 |  | Viridiflorol | 1607 | 1592 | KI, MS | 0.49 ± 0.03 |
| **36** |  | **Khusimone** | **1623** | **1604** | **KI, MS** | **3.10 ± 0.10** |
| 37 |  | 1,10-di-epi- Cubenol | 1633 | 1619 | KI, MS | 0.65 ± 0.02 |
| 38 |  | Eremoligenol | 1650 | 1631 | KI, MS | 0.98 ± 0.07 |
| 39 |  | allo-Aromadendrene epoxide | 1655 | 1641 | KI, MS | 0.17 ± 0.01 |
| 40 |  | epi-α-Muurolol | 1657 | 1642 | KI, MS | 0.68 ± 0.03 |
| **41** |  | **β-Eudesmol** | **1670** | **1650** | **KI, MS** | **2.52 ± 0.24** |
| **Table 3.** *(Continued)* | | | | | | |
| No |  | Name | KI^a^ | KI^b^ | Identification | % |
| **42** |  | **epi-Zizanone** | **1685** | **1670** | **KI, MS** | **1.77 ± 0.21** |
| **43** |  | **Cadalene** | **1690** | **1676** | **KI, MS** | **1.60 ± 0.05** |
| **44** |  | **Khusinol** | **1695** | **1680** | **KI, MS** | **3.77 ± 0.19** |
| **45** |  | **Zizanal** | **1703** | **1697** | **KI, MS** | **3.26 ± 0.01** |
| **46** |  | **8-Cedren-13-ol** | **1718** | **1689** | **KI, MS** | **2.04 ± 0.06** |
| 47 |  | (Z)-5-hydroxy-Calamenene | 1730 | 1713 | KI, MS | 0.16 ± 0.03 |
| **48** |  | **Vetiselinenol** | **1745** | **1731** | **KI, MS** | **3.41 ± 0.19** |
| **49** |  | **Khusimol** | **1772** | **1742** | **KI, MS** | **23.78 ± 0.13** |
| **50** |  | **(E)-Isovalencenol** | **1814** | **1793** | **KI, MS** | **6.79 ± 0.12** |
| 51 |  | Nootkatone | 1819 | 1806 | KI, MS | 0.99 ± 0.03 |
| **52** |  | **β-Vetivone** | **1844** | **1823** | **KI, MS** | **2.14 ± 0.00** |
| **53** |  | **α-Vetivone** | **1871** | **1843** | **KI, MS** | **3.84 ± 0.04** |

^a^ Kováts retention indices calculated against C_7_–C_30_ *n*-alkanes on nonpolar Elite–5 column.

^b^ Kováts retention indices on nonpolar DB–5 column reported in literature (Adams, 2009).
